# Atraumatic restorative treatment compared to the Hall Technique for occluso-proximal carious lesions in primary molars; 36-month follow-up of a randomised control trial in a school setting

**DOI:** 10.1186/s12903-020-01298-x

**Published:** 2020-11-11

**Authors:** Mariana Pinheiro Araujo, Nicola Patricia Innes, Clarissa Calil Bonifácio, Daniela Hesse, Isabel Cristina Olegário, Fausto Medeiros Mendes, Daniela Prócida Raggio

**Affiliations:** 1 Department of Paediatric Dentistry, School of Dentistry, University of São Paulo, São Paulo, Brazil; 2 School of Dentistry, University of Dundee, Dundee, Scotland UK; 3 School of Dentistry, Cardiff University, Heath Park, Cardiff, CF14 4XY UK; 4 Department of Paediatric Dentistry, Academic Centre for Dentistry Amsterdam (ACTA), Amsterdam, The Netherlands; 5 Department of Public and Child Dental Health, Dublin Dental University Hospital, Trinity College, Dublin, Ireland UK

**Keywords:** Hall Technique, Atraumatic Restorative Treatment, Restoration, Primary molars, Dental caries, Management, Randomized controlled trial, Non-AGPs

## Abstract

**Background:**

Atraumatic Restorative Treatment (ART) and the Hall Technique (HT) are both minimally invasive, non-aerosol generating procedures (non-AGPs). They seem to have never been directly compared, nor has the HT been studied in a non-clinical setting. This study compared the HT and ART restorations placed in a school setting after 36 months.

**Methods:**

Children (5–10 yo) who had a primary molar with a dentinal occluso-proximal, cavitated carious lesion were allocated to the ART (selective removal) or HT arms. Primary outcome: restoration survival over 36-months (using Kaplan–Meier survival analysis, log rank test, and Cox regression). Secondary outcomes: (1) occlusal vertical dimension (OVD) (1, 2, 3, 4 weeks) and (2) child self-reported discomfort; (3) treatment acceptability (immediately following interventions); (4) Child Oral Health Related Quality of Life (OHRQoL), before treatment and after 6 months and (5) a post hoc analysis of time to tooth exfoliation (1, 6, 12, 18, 24, 30, 36 months).

**Results:**

One-hundred and thirty-one children (ART = 65; HT = 66) were included (mean age = 8.1 ± 1.2). At 36 months, 112 (85.5%) children were followed-up. Primary outcome: restoration survival rates ART = 32.7% (SE = 0.08; 95% CI 0.17–0.47); HT = 93.4% (0.05; 0.72–0.99), *p* < 0.001; Secondary outcomes: (1) OVD returned to pre-treatment state within 4 weeks; (2) treatment discomfort was higher for the HT (*p* = 0.018); (3) over 70% of children and parents showed a high acceptability for treatments, with crown aesthetics being a concern for around 23% of parents; (4) Child OHRQoL improved after 6 months; and (5) teeth treated with the HT exfoliated earlier than those in the ART group (*p* = 0.007).

**Conclusions:**

Both ART and the HT were acceptable to child participants and their parents and all parents thought both restorations protected their child’s tooth. However, the crown appearance concerned almost a quarter of parents in the HT arm. Children experienced less discomfort in the ART group. Although both treatments can be performed in a non-clinical setting and have the advantage of being non-aerosol generating procedures (non-AGPs), the HT had almost three times higher survival rates (93.4%) for restoring primary molar occluso-proximal cavities compared to ART (32.7%).

**Trial registration:**

This trial was registered in ClinicalTrials.gov (NCT02569047), 5th October 2015. https://clinicaltrials.gov/ct2/show/study/NCT02569047?cond=Hall+Technique+Atraumatic+Rest orative+Treatment&draw=2&rank=2.

## Background

In Brazil, there is still a high dental treatment need for children, with approximately 80% of them experiencing untreated carious lesions in the primary dentition [1]. The most common intervention for dental caries continues to be conventional restorative treatment [2] where carious dentine is removed with rotary instruments and the cavity filled with composite resins. Treatment can improve children’s quality of life as well as that of their families [3, 4]. The most common intervention for dental caries continues to be conventional restorative treatment [2] where carious dentine is removed with rotary instruments and the cavity filled with composite resins. Although the origins of dental anxiety are multifactorial [5, 6], the use of rotary instrument and local anaesthesia during dental treatment has been found to contribute to negative experiences and affect behaviour, increasing dental fear and anxiety in future dental appointments [7–9].

Minimally invasive dentistry (MID) as an approach, should be the standard care for managing carious lesions [10], slowing the downward restorative spiral and reducing discomfort during the treatment [11, 12]. Approaches such as the Atraumatic Restorative Treatment (ART) and the Hall Technique (HT), neither of which require local anaesthetic nor the use of rotary instruments, fit in with MID principles.

ART [12, 13], using only hand instruments to remove carious tissue and prepare the cavity, has been commonly used in paediatric dentistry because it is associated with lower levels of anxiety, pain and discomfort, as well as greater acceptance by children, compared to the conventional treatment [14–17]. In addition, ART can be delivered, without electricity, running water or rotary instruments and can be used in dental clinics and communities where access to dental equipment is limited. Good survival rates have been reported for single-surface cavities in both the primary and permanent dentitions [18–21]. However, when used to restore multi-surface cavities, ART has shown lower survival rates, ranging from 93 to 12.2% of success after 3 years [18, 19, 21, 22].

The HT [23] involves placing a preformed metal crown over a carious tooth using glass ionomer cement (GIC) [24]. No tooth preparation or carious tissue removal is required eliminating the need for rotary instruments and local anaesthetic. The HT has become routinely used in many countries and is currently recommended in the American Academy of Pediatric Dentistry [25] and SDCEP guidelines [26]. The technique has lower or similar levels of discomfort when compared to conventional treatments [27–29]. However, child self-reported discomfort has only been assessed in one study [27], showing no difference between children treated with conventional restorations using local anaesthetic, HT and non-restorative cavity control. The HT has been reported to have high success rates (over 90% up to 5 years follow-up) for restoring multi-surface lesions in children [30, 31]. However, there are no trials comparing the HT with different approaches in a non-clinical setting for treating children where dental facilities are not available.

This randomised clinical trial aims to compare tooth level restoration survival at 36 months (primary outcome) for ART and the HT carried out in a school setting to manage occluso-proximal carious lesions in primary molars [32]. Secondary outcomes are: OVD resolution after the crown is placed using the HT; child reported discomfort related to the treatment; children and their parents’ acceptance after the treatments; and child’s OHRQoL.

## Material and methods

### Trial design

This is a two-arm, parallel group, patient-randomised controlled, superiority trial with a 1:1 allocation ratio.

The protocol [32] set the age range for children to be included in this study from 6 to 8 years old. However, there were not enough children within that age group who fitted the inclusion criteria at the schools, so the age range was increased to include five to ten-year olds.

### Ethical aspects

This study was approved by the Research Ethics Committee of the Dental School of the University of São Paulo (protocol number 1.293.935), registered in ClinicalTrials.gov (NCT02569047) and written according to CONSORT guidelines for randomised controlled trials. Participants were included after their parents/carers were given detailed information about the objectives and procedures of this trial and had given written consent for their children to participate. Eligible children had the trial and treatments explained to them and were invited to accept or decline to participate using an assent form as their willingness to participate in the study.

### Deviations from protocol

It was planned that OHRQoL would be assessed through questionnaires to child participants and their parents/carers. However, the response of parents/carers to the OHRQoL questionnaire before and 6 months after the treatments was less than 50%. This was likely to be a biased sample, so the results of parents/carers questionnaires are not reported. Perceptions and concerns related to the tooth appearance will not be reported as the questionnaire was related to child’s whole mouth and complete smile and did not apply to a single tooth. The outcome related to the cost-effectiveness analysis will be reported elsewhere and will consider the restoration survival rate reported in this article. A *post-hoc* analysis of tooth exfoliation was carried out as during the data collection a difference was observed. This outcome was not previously planned to be collected.

### Sample size

The sample size calculation was based on the primary outcome—restoration survival after 36 months, defined as the absence of minor and major failures (Table 1) using the log-rank test and survival analysis. This involved a two-tailed test based on survival rate reported for occluso-proximal ART restorations of 62%, obtained from a previous study after 2 years follow-up [33], using the absolute difference of 25% between groups, significance level of 80%. This gave an estimate of 103 children to be recruited with one tooth each treated within the study. After increasing by 20% to compensate possible loss to follow-up, the final sample size was set at minimum 124 children (62 participants per group).

**Table 1:** Evaluation criteria for restorations assessments ( adapted from Innes et al. 2007) [29]

Outcome	Outcome criteria	
	ART	Hall Technique
Success	Satisfactory restoration, no intervention required No signs or symptoms of pulp damage Tooth exfoliated with no minor or major failures	Satisfactory crown, no intervention required No signs or symptoms of pulp damage Tooth exfoliated with no minor or major failures
Minor failures	New carious lesions (around the restoration or in the tooth) Restoration fracture or wear—intervention is required (> 0.5 mm) Restoration loss—tooth can be re- restored Reversible pulpitis—can be managed without the need of pulpotomy or extraction	Crown perforation Crown loss—tooth can be re-restored Reversible pulpitis—can be managed without the need of pulpotomy or extraction
Major failures	Irreversible pulpitis, dental abscess or fistula—requires pulpotomy or extraction Restoration loss—tooth cannot be re- restored Tooth fracture	Irreversible pulpitis, dental abscess or fistula—requires pulpotomy or extraction Crown loss—tooth cannot be re- restored Tooth fracture

### Randomisation

Allocation sequence was generated electronically using a website (https://randomization.com/) with permuted block sizes of 4, 6 and 8, stratified by operator and sealed in sequentially numbered opaque envelopes.

Randomisation was at participant level, with children allocated to either ART (control group) or HT (intervention group) and one of the operators (specialist, student 1 or student 2). Children were enrolled and randomly allocated using the previous generated allocation sequence by an independent dentist that was not involved with the treatments. The envelopes were selected sequentially by the dentist and opened when the child presenting all the inclusion criteria was ready to have the treatment initiated by one of the operators, as described in this trial’s protocol [32].

### Blinding

Blinding operators, children, parents and the outcome assessor was not possible as both treatments use different techniques and distinct materials. Also, the restoration appearances are not similar, being possible to identify the group allocation based on material’s appearance.

### Participants

Children from 5 to 10 years old attending public schools in the city of Tietê, Brazil, were screened and invited to participate in this study if they presented with:

- at least one dentinal occluso-proximal cavitated carious lesion in a primary molar with no signs or symptoms of pulp involvement;
- generally cooperative behaviour that could be managed by the operators in the school setting; and
- no known medical conditions.

Children eligible to participate in this study received an envelope to take home for their parents/carers containing an information sheet about the trial and a parents/carers’ informed consent form. If parents/carers were interested in their children taking part in the trial, they sent the consent form signed back to the school before the child’s treatment commencement. At the time of the treatment, children whose parents/carers agreed to take part in this trial received an assent form asking if they also agreed to take part. In cases where the child had more than one cavity eligible for inclusion in the study, only one cavity was selected following the procedures described on the protocol [32].

### Trial setting

The trial was set in the public schools of Tietê, a countryside city in the state of São Paulo, Brazil. Treatments and clinical assessments were carried out in schools’ classrooms, with no dental facilities such as a dental chair, access to radiographic investigation, rotary instruments, suction equipment or air-drying.

The outcome assessor performed the follow-up examinations and questionnaires assessments in empty classrooms at the schools.

### Interventions

Children were treated during school hours in empty classrooms, lying on a school table with a mattress. The operators were positioned at the end of the table sitting on a chair high enough to access the child’s mouth and used a light attached in their forehead to enable visualisation of the child’s mouth.

Both treatments were carried out according to standard protocols [13, 24]. In the control group (ART) cavities were prepared using hand instruments for selective carious tissue removal and restored using the encapsulated high viscosity GIC EQUIA Forte (GC Corp., Leuven, BE). In the intervention group (HT) cavities had no carious tissue removal, nor tooth preparation/reduction to facilitate the crown fitting or crown trimming. An orthodontic separator was placed between the tooth when there was a tight proximal contact point between the tooth to be fitted with crown and the adjacent tooth for a period between one and seven days, depending on the children’s physiological response. Preformed metal crowns (3M/ESPE, St Paul, USA) were cemented using encapsulated GIC Fuji I (GC Corp., Leuven, BE). Detailed information on how interventions were carried out is published elsewhere [32] and also available in Additional files 1 and 2.

### Recruiting, operating and assessing staff

Two trained and calibrated specialists in Paediatric Dentistry screened children at the schools to assess their eligibility for the trial.

Three operators carried out the interventions: one experienced specialist in Paediatric Dentistry and two final-year undergraduate dental students. All operators were trained for both treatments by experienced clinicians who were familiar with the techniques. The undergraduate students also underwent a 2-week training period in a school setting under the supervision of experienced clinicians. Participants treated during this period were children who matched the inclusion criteria and whose parents/carers had formally consented to participate in this trial. These children were not included in the final study sample.

The outcome assessor was a dentist experienced in treating children who was not involved with the treatments. Training consisted of a visual lecture and laboratory training with extracted restored teeth for assessing the treatment outcomes according to the agreed evaluation criteria. The clinical evaluations of children included were carried out at 1, 2 and 3 weeks and 1, 6, 12, 18, 24, 30 and 36 months. Intra-rater reliability was checked by 20% of the sample size that were evaluated at 1-week follow-up being re-evaluated after 2 weeks and analysed using a kappa test.

### Trial outcomes

#### Restoration survival at 36 months (primary outcome)

Clinical outcomes related to restoration survival were evaluated at 1, 6, 12, 18, 24, 30 and 36 months. The definitions of “Success”, “Minor Failure” and “Major Failure” outcomes are reported in Table 1 (adapted from Innes et al. 2007) [29]. At the follow-up appointments, each tooth/restoration could only be scored as “successful” or having experienced a “Minor” or “Major failure”. In cases where the same tooth presented both Minor and Major failures, the Major failure was recorded as the outcome.

#### Occlusal vertical dimension (OVD) resolution

OVD was assessed only in the HT group by modifying the method reported by van der Zee and van Amerongen [34]. It was assessed before and after the treatments and at the subsequent follow-ups (at 1, 2, 3 and 4 weeks after the crown was placed) using a millimetre dental probe (University North Carolina CP15).

**Fig. 1:**
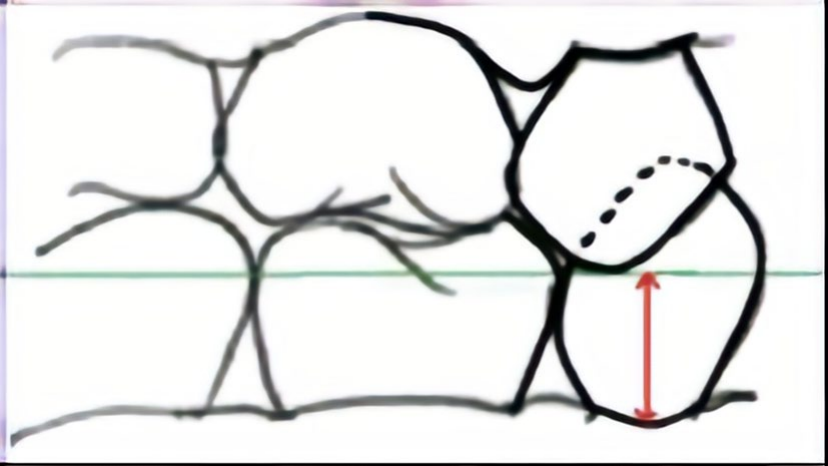
Method for measuring the OVD of children in the HT group [32] adapted from van der Zee and van Amerongen [34]

The measurements were carried out using the canines on the same side the treatment was performed. In case children had the canines on the same side of the treatment missing, the contralateral canines were used to measure the OVD. If none of the canines were present in mouth, the measurements were carried out using the first primary molars. Children’s OVD measurement was recorded using the distance from the lowest point of the gingiva of lower canine to the upper canine tip (Fig. 1). Children in the ART group did not have the OVD recorded before and after the treatment. The restoration was trimmed to accommodate the child occlusion using articulating paper.

#### Discomfort at time of intervention

**Fig. 2:**
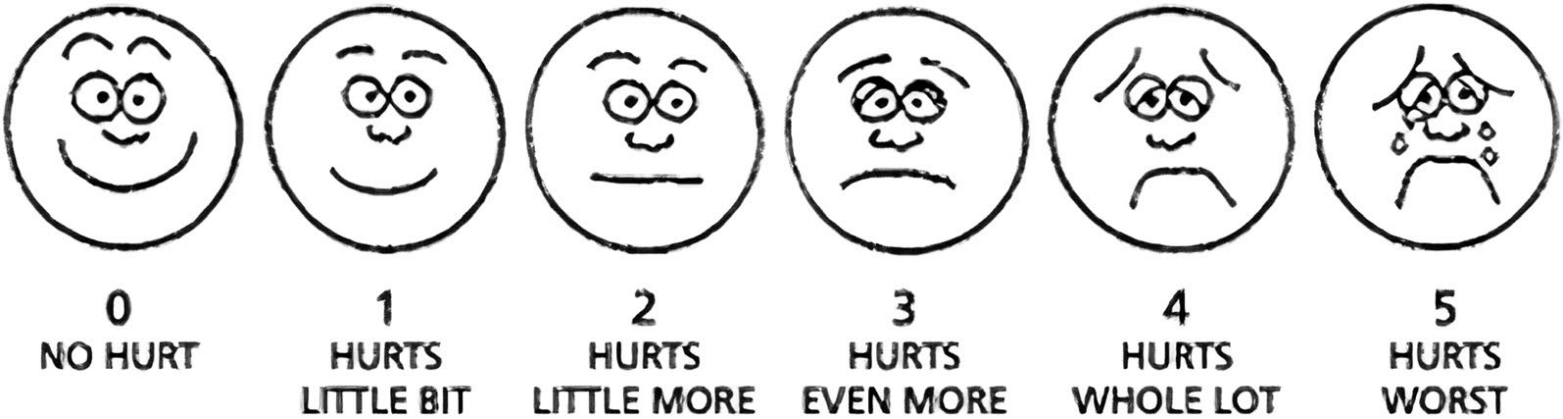
Wong-Baker Faces Pain Scale [35] used to measure children’s self-reported discomfort level during the intervention

The Wong-Baker Faces Pain Scale (WBFPS), with six numbered faces from 0 to 5 (Fig. 2 [35], was used to assess the child’s reported level of discomfort before and after treatment for both groups (ART and HT). For the HT, discomfort was also recorded before and after placement of orthodontic separators. An outcome assessor (not involved in the child’s treatment) described the scale to the child in an area where the operator was not present.

Children were asked to rate their discomfort level by pointing to the face on the scale that they thought represented them during their treatment and the outcome assessor recorded it. Pre- treatment scores were checked for similarity between the groups at baseline (to verify randomisation). Only post-treatment scores were analysed statistically.

#### Treatment acceptability

##### Children

To evaluate treatment acceptability, a modified version of Bell et al. 2010 [28] (five questions with a face-illustrated and text Likert scale: strongly agree, agree, no opinion, disagree, and strongly disagree) translated to Portuguese was used (see Additional file 3). The outcome assessor interviewed each child using the proposed questions immediately after treatment in a separate room from where the treatment was performed and from the operators.

#### Parents

The questionnaire consisted of five questions and text Likert scale with five possible answers: strongly agree, agree, no opinion, disagree, and strongly disagree (see Additional file 4). Children took the parents/carers’ questionnaires home after treatment. Parents/carers returned completed questionnaires to the school.

#### Oral Health Related Quality of Life (OHRQoL)

This was assessed through the CPQ8-10 (Child Perceptions Questionnaire) [36] through an interview with the children by the outcome assessor immediately before the intervention and after 6 months. There were 25 questions in four domains: Oral Symptoms, Functional Limitations, Emotional Well-Being and Social Well-Being, with five possible responses: never (0), once or twice (1), sometimes (2), often (3) and every day or almost every day (4).

The final CPQ8-10 score was the summation of the questionnaire scores. The higher the score, the worse the child’s quality of life was when the questionnaire was applied. Scores were also considered within each domain.

#### Teeth exfoliation

Data related to exfoliation of the treated tooth were collected for both groups at 1, 6, 12, 18, 24, 30 and 36 months. Teeth included in the study were marked as present or absent at the time of examination. If the tooth included was absent at any time-point, the child was asked if the tooth had exfoliated or was extracted by another dentist not involved in the present trial.

Children who presented a Major failure related to pulp involvement in the tooth included in the study (Table 1) were not included in the exfoliation analysis, as the Major failure might have interference on the exfoliation time (root/bone resorption around the tooth).

### Statistical analysis

Microsoft Windows Excel 2013 was used for data entry and Stata 13.0 for data analysis. Quantitative variables had the normality checked by Kolmogorov–Smirnov test. The significance level of 0.05 was assumed for all statistical analyses.

#### Restoration survival at 36 months (primary outcome)

Kaplan–Meier survival analysis and log-rank test were carried out to analyse restorations’ survival rate. Cox regression test investigated associations between survival and the other variables; operator (with/without experience), age, sex (male/female), dmft/DMFT, jaw (upper/lower), side (right/left), tooth (1st/2nd primary molar), cavity volume and moisture control when the restoration was being performed (no saliva or gingival bleeding). Hazard ratio (HR) and 95% Confidence Interval (95% CI) were derived. The intra-rater reproducibility for restoration evaluation was calculated using the weighted kappa test.

#### Occlusal vertical dimension (OVD) resolution

Descriptive analysis was considered using average mean and standard deviation (SD). Multilevel linear regression (95% CI) was carried out to analyse when the OVD was re-stablished and if there was any association with other variables as age, tooth (1st/2nd primary molar) and jaw (upper/lower).

#### Discomfort at time of intervention

As discomfort was measured twice for the HT group (after orthodontic separator and after crown cementation), the data were analysed and reported in two ways: (1) using the higher score given by the children of the two moments of discomfort measurement (orthodontic separator or crown cementation) to show the overall discomfort experience; and (2) using only the score for discomfort after the crown cementation to allow comparability with other studies. For the evaluation and association of the final discomfort between the groups and other variables ordered logistic regression (95% CI) was used. Both univariate and adjusted analysis are reported in this paper.

#### Treatment acceptability (child and parent)

These were reported using descriptive statistics. Data for missing questions were not imputed and only completed questionnaires were analysed. The number of responses and missing data and their distribution were reported.

#### Oral health related quality of life

For statistical analysis, only children who answered the questionnaire at the baseline and after 6 months were considered. Wilcoxon test was carried out for paired samples (before and after the treatment). Mann–Whitney test was carried out to compare data between groups (unpaired).

#### Teeth exfoliation

Kaplan–Meier survival analysis and log-rank test were carried out to analyse teeth exfoliation. Cox regression investigated associations between the exfoliation and the other variables; age, sex (male/female), jaw (upper/lower), side (right/left), tooth (1st/2nd primary molar) and cavity volume. Hazard ratio (HR) and respective 95% CI were derived.

### Data monitoring

There was no external Data Monitoring Committee and independent oversight of trial data collection and management were undertaken by MPA. The Chief Investigator (DPR) had overall responsibility of the study and was the data custodian.

## Results

### Screening and recruitment

There were 1258 children screened at seven public schools in Tietê in October 2015 with 214 being found to be potentially eligible and having invitations to participate sent to their parent/carers.

Treatments were carried out from October to December 2015. The children were assigned using random allocation with the aid of a randomisation list to one of the three operators with them treating similar numbers (44, 44 and 43) of participants.

Outcome assessor’s weighted kappa value for intra-rater reproducibility was 0.93.

### Participants and interventions

**Fig. 3:**
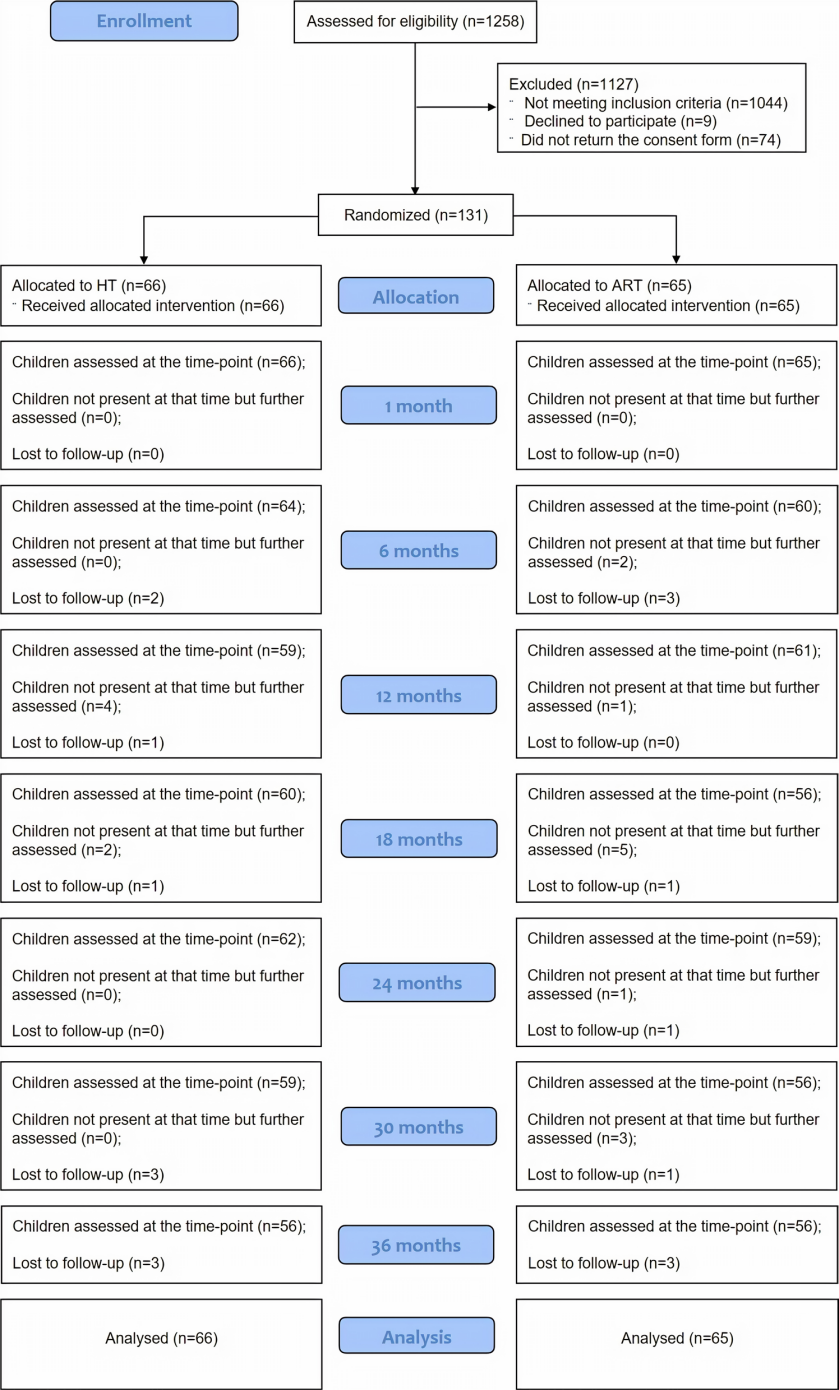
CONSORT flow diagram of participants' progress through trial phases

Out of 214 children invited to participate, 131 (61%) were consented, randomised and had treatment carried out in this trial. Sixty-six children (50.4%) were assigned to the HT group and 65 (49.6%) to the ART group. The CONSORT flow diagram (Fig. 3) shows the participants’ progress through the trial phases.

Participant’s baseline characteristics were similar between the groups related to sex, age, dmft/DMFT and tooth treated (*p* > 0.05). Further information for participant’s baseline characteristics and a schedule of outcomes assessments are available in the Additional files 5 and 6 respectively.

### Outcomes assessments

#### Restoration survival at 36 months (primary outcome)

One-hundred and twelve children (85.5%) had the study tooth evaluated after 36 months and 19 children (14.5%) were lost to follow-up. Children not present or lost to follow-up were censored and therefore, data was considered in the analysis.

**Fig. 4:**
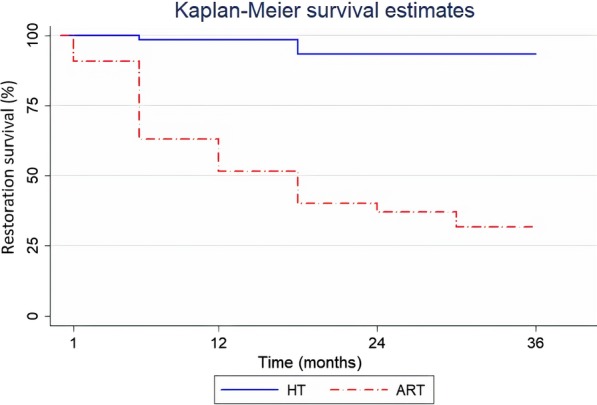
Kaplan–Meier Survival curves over 36 months with follow-up data collected every 6 months for ART and HT (n = 131)

After 36 months, the restoration survival rates were: ART = 32.7% (SE = 0.08; 95% CI 0.17–0.47) and HT = 93.4% (SE = 0.05; 95% CI 0.72–0.99), *p* < 0.001 calculated by log rank test. The Kaplan–Meier survival curves are shown in Fig. 4. Failures at 36-month follow-up are described in Table 2 and 6-monthly survival rates for both groups are presented in Additional file 7.

**Table 2:** Treatment failures by type and arm at 36-month follow-up (n = 131)

	Outcome criteria			
	Success	Minor failure	Major failure	Lost to follow-up
ART	23 (35%)	28 (43%)^a,b^	5 (8%)^d^	9 (14%)
HT	54 (82%)	1 (1.5%)^c^	1 (1.5%)^d^	10 (15%)

^a^Restoration fracture/wear ≥ 0.5 mm—intervention required = 4 (6%)

^b^Restoration loss—tooth can be re-restored = 24 (37%)

^c^Crown loss—tooth can be re-restored

^d^Irreversible pulpitis, dental abscess or fistula—requires pulpotomy or extraction

Cox Regression found no association between restoration survival and other variables with the ART as the reference group (Table 3). Stratified analysis was carried out to investigate if any of the variables were associated with failures within the groups and no tendency to association was observed.

**Table 3:** Univariate and adjusted Cox regression analysis for restoration survival (36-month follow-up)

Variable	Success n (%)	Failure n (%)	Total (n)	HR univariate^†^ 95% CI^‡^	*p* value	HR adjusted^†^ 95% CI^‡^	*p* value
*Group*							
ART (ref)	32(49.23)	33 (50.77)	65				
Hall Technique	64 (96.97)	2 (3.03)	66	0.052 0.013–0.22	< 0.001*	0.058 0.014–0.24	< 0.001*
*Operator*							
Specialist (ref)	35 (79.55)	9 (20.45)	44				
Student 1	29 (67.44)	14 (32.56)	43	1.67 0.72–3.86	0.233	-	-
Student 2	32 (72.73)	12 (27.27)	44	1.20 0.60–2.85	0.682	-	-
*Age (years)*							
5–6.9 (ref)	16 (66.67)	8 (33.33)	24	1.33 0.67–3.12	0.510	-	-
7–8.9	55 (73.33)	20 (26.67)	75	1.39 0.48–4.02	0.538	-	-
≥ 9	25 (78.13)	7 (21.88)	32				
*Sex*							
Male (ref)	57 (71.25)	23 (28.75)	80				
Female	39 (76.47)	12 (23.53)	51	0.86 0.43–1.73	0.673	-	-
*dmft/DMFT*							
1—2	29 (61.70)	18 (38.30)	47				
3—4	34 (75.56)	11 (24.44)	45	0.53 0.25–1.13	0.102	0.63 0.29–1.36	0.242
≥ 5	32 (84.21)	6 (15.79)	38	0.33 0.13–0.84	0.019*	0.44 0.17–1.16	0.097
*Jaw*							
Upper (ref)	57 (72.15)c	22 (27.85)	79				
Lower	39 (75.00)	13 (25.00)	52	0.86 0.43–1.71	0.668	-	-
*Side*							
Right (ref)	48 (68.57)	22 (31.43)	70				
Left	48 (78.69)	13 (21.31)	61	0.62 0.31–1.23	0.170	0.54 0.26–1.10	0.089
*Tooth*							
1st primary molar (ref)	62 (75.61)	20 (24.39)	82				
2nd primary molar	34 (69.39)	15 (30.61)	49	1.19 0.610–2.334	0.605	-	-
Cavity volume**							
0–10 mm^3^ (ref)	46 (73.02)	17 (26.98)	63				
11–20 mm^3^	31 (75.61)	10 (24.39)	41	0.94 0.43–2.06	0.882	-	-
21–30 mm^3^	16 (76.19)	5 (23.81)	21	0.98 0.36–2.65	0.962	-	-
> 30mm^3^	3 (60.00)	2 (40.00)	5	1.39 0.32–6.06	0.657	-	-
*Moisture control (no saliva or gingival bleeding contamination)*							
Maintained (ref)	95 (74.22)	33 (25.78)	128				
Not maintained	1 (33.33)	2 (66.67)	3	3.161 0.75–13.36	0.118	2.24 0.48–10.51	0.305
Total	96 (73.28)	35 (26.72)	131				

^†^*HR* Hazard ratio, ^‡^*CI* confidence interval

^*^Indicates statistically significance differences (*p* < 0.05)

^**^One child in the ART group did not have the cavity dimensions measured and recorded by the operator

#### OVD resolution

**Fig. 5:**
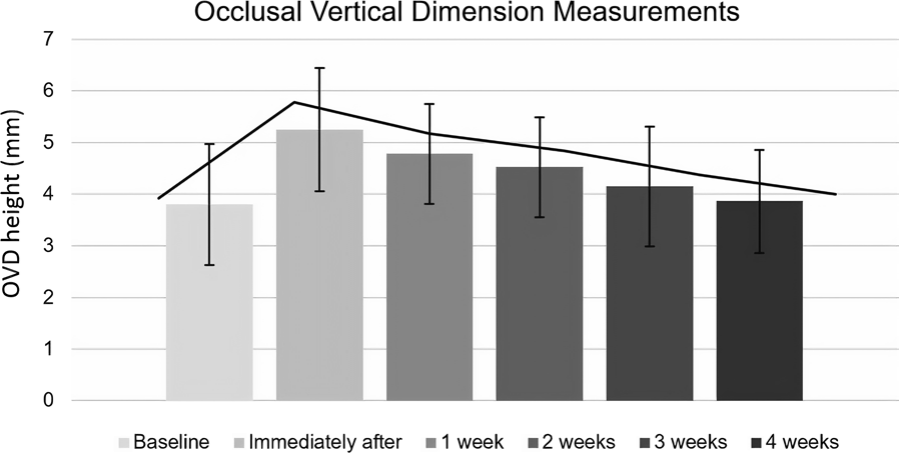
OVD measurements from one to four weeks for the HT group

OVD was only measured in children in the HT group (n = 66). The mean OVD at baseline was 3.80 mm (SD ± 1.17 mm); immediately after crown placement it was 5.25 mm (SD ± 1.20), an average increase of 1.45 mm (SD ± 0.87 mm). Multilevel linear regression showed that the OVD returned to its pre-crown measurements within four weeks after treatment. There was no difference in OVD measurements at baseline and four weeks after treatment (*p* = 0.057) (Fig. 5).

#### Discomfort at time of intervention

**Fig. 6:**
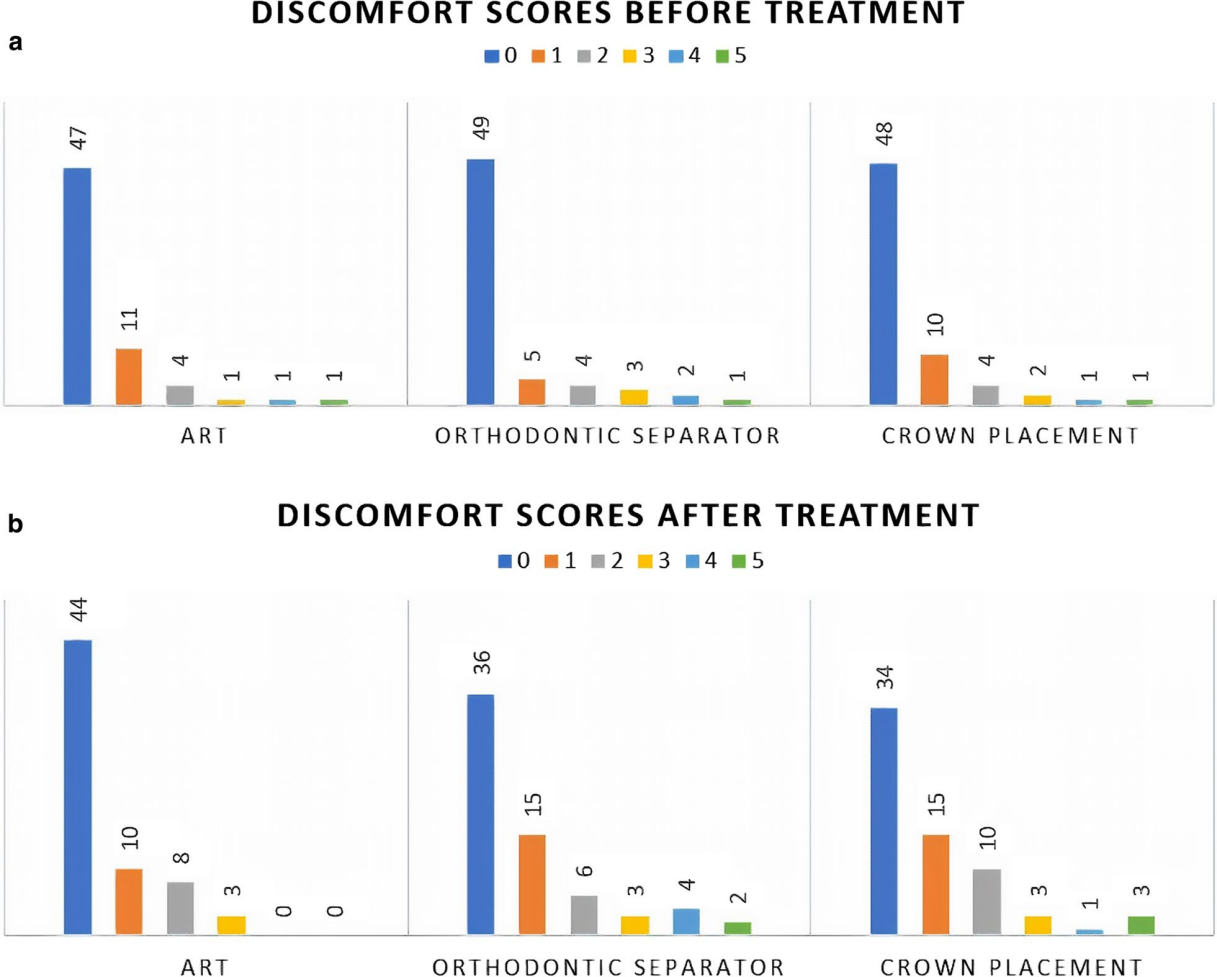
**a** WBFPS scores’ distribution between the groups (0 = no discomfort to 5 = maximum score for discomfort) at baseline. **b** WBFPS scores’ distribution between groups (0 = no discomfort to 5 = maximum score for discomfort) after treatment

There was no association between child reported discomfort scores before and after the interventions (IRR = 0.98, CI 0.82–1.17, *p* = 0.819) and no differences between the ART and HT groups at the baseline (Fig. 6a and Fig. 6b respectively).

Taking the highest discomfort score between the orthodontic separator placement and crown cementation for the HT group, discomfort level was statistically significantly higher than the ART group (*p* = 0.001). Table 4 shows the ordered logistic regression analysis.

**Table 4:** Ordered Logistic Regression analysis of the final discomfort between the groups and independent variables considering the highest discomfort score between the orthodontic separator and the crown placement

Variables	Unadjusted OR (95% CI)	*p-*value	Adjusted OR (95% CI)	*p-*value
*Treatment*				
ART (ref)				
HT	3.20 (1.62–6.32)	0.001*	3.67 (1.79–7.49)	< 0.001*
*Age (years)*				
5–6.9 (ref)				
7–8.9	0.67 (0.28–1.60)	0.365	0.70 (0.27–1.79)	0.454
≥ 9	0.93 (0.35–2.49)	0.888	0.85 (0.29–2.49)	0.770
*Sex*				
Male (ref)				
Female	0.95 (0.49–1.85)	0.887		
*Operator*				
Specialist (ref)				
Student 1	0.88 (0.39–1.98)	0.756		
Student 2	1.61 (0.72–3.59)	0.246		
*Jaw*				
Upper (ref)				
Lower	1.32 (0.68–2.55)	0.417		
*Primary Tooth*				
1st Molar (ref)				
2nd Molar	0.53 (0.27–1.05)	0.068	0.53 (0.25–1.09)	0.086
*DMFT/dmft*				
0 and 1 (ref)				
3 and 4	0.96 (0.44–2.05)	0.907	0.93 (0.42–2.06)	0.854
≥ 4	0.54 (0.24–1.25)	0.150	0.43 (0.25–1.09)	0.086

*ART* Atraumatic Restorative Treatment, *HT* Hall Technique, *OR* odds ratio, *95% CI* 95% confidence interval

^*^Statistically significant difference (*p* < 0.05)

Discomfort scores following crown placement (i.e. not considering the orthodontic separator score) showed no significant difference between the groups (*p* = 0.055). Considering other variables in the adjusted model, discomfort after crown placement was significantly higher in the HT group and influenced by children’s age and dmft/DMFT (*p* = 0.025). The two models using ordered logistic regression for this analysis can be found in Additional file 8.

For discomfort levels in the HT group, 34 children (51.5%) reported the same discomfort score for separator placement and crown cementation, 11 children (16.7%) reported a higher level of discomfort after the orthodontic separator and 18 children (27.3%) reported a higher level of discomfort after the crown cementation. Three children (4.5%) did not need the orthodontic separator placement as there was enough interproximal space to fit the crown. There was no evidence of a difference between the final discomfort after the orthodontic separator placement and after the crown cementation (IRR = 1.01, CI 0.63–1.65, *p* = 0.948).

#### Treatment acceptability

a. Children

**Fig. 7:**
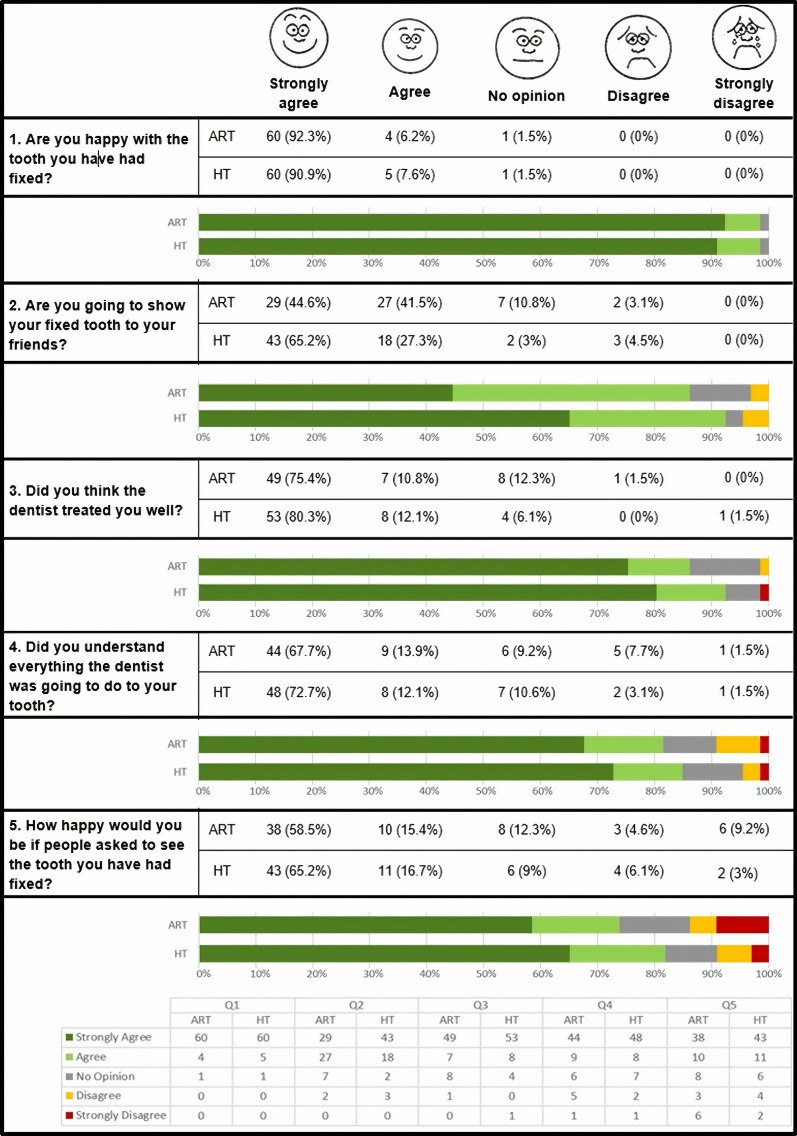
Distribution of children’s responses to the 5 questions investigating treatment acceptability for ART and HT. Based on Bell et al. 2010 [28] (n = 131)

There was 100% completion rate. For both groups, over 70% of responses were “strongly agree” or “agree” with all positive statements, increasing to over 85% when “no opinion” was considered for each question (Fig. 7).

The greatest differences between the groups were seen with the number of total disagreements in questions 4 (ART = 6/HT = 3) and 5 (ART = 9/HT = 6).

b. Parents

**Fig. 8:**
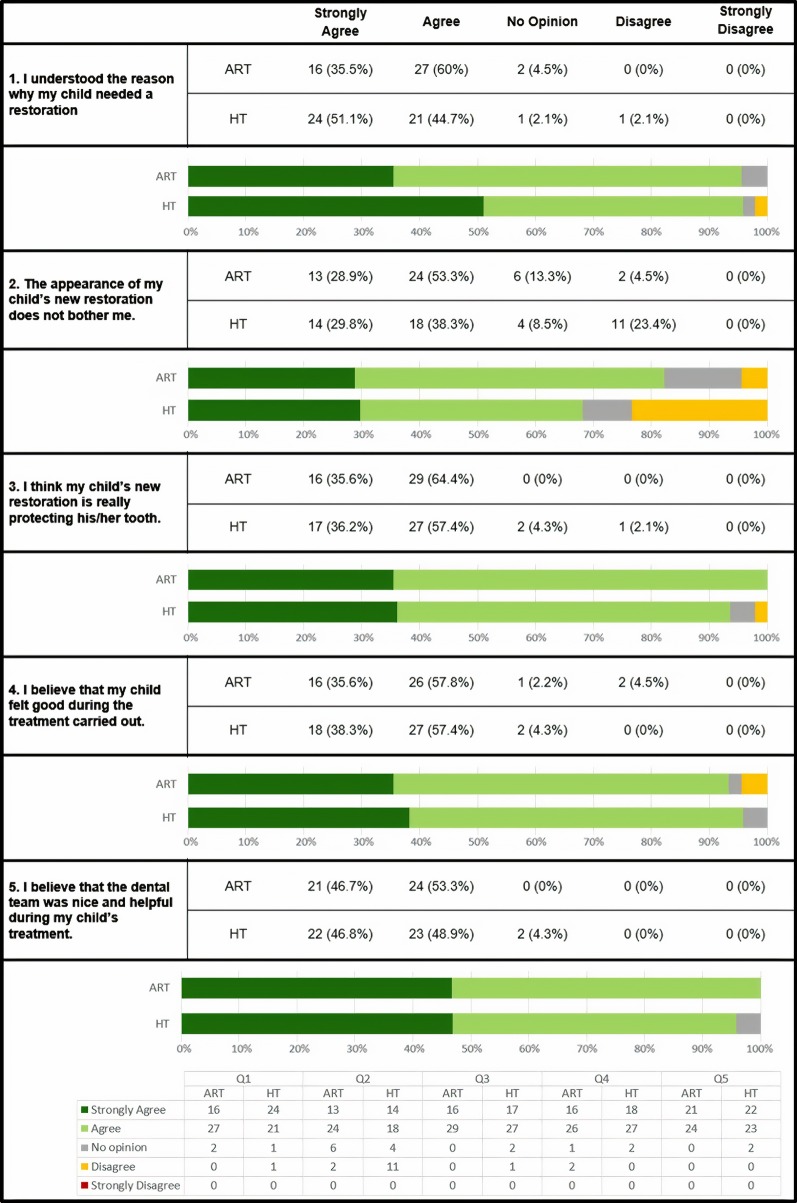
Distribution of parents’ responses to the 5 questions investigating treatment acceptability for ART and HT (ART n = 45/65; HT n = 47/66)

Parental response rate for treatment acceptability questionnaire was 70.23% (n = 92). The percentage of the answers “strongly agree” and “agree” was over 70% for all statements with a similar distribution between groups (Fig. 8). The only difference was for “The appearance of my child’s new restoration does not bother me”, where 23.4% of the parents in the HT group disagreed with the statement compared to 4.5% in the ART group.

#### Oral Health Related Quality of Life (CPQ 8–10)

All 131 children completed the questionnaire at baseline and 93.9% (n = 123) at 6-month follow-up. There was evidence of a significant improvement in OHRQoL for both total score and domains (*p* < 0.05), apart from Oral Symptoms in the ART group where there was no difference at baseline or at 6 months (*p* = 0.052). There was no evidence of a difference for total scores or individual domains between ART and HT groups (*p* > 0.05). Table 5 shows the comparison between baseline and 6-month follow-up, change scores and effect sizes.

**Table 5:** Total and individual domain scores, changes in scores and effect sizes for Child Perceptions Questionnaire (CPQ8-10) at baseline and 6-month follow-up (n = 123)

	Baseline	6 m follow-up	Changes in scores	
	Mean (SD)	Mean (SD)	Mean (SD)	Effect size
*ART (n* = *59)*				
Oral symptoms	5.88 (3.68)^▲^	5.02 (3.75)^▲^	0.86 (3.58)	0.23
Function limitations	5.00 (4.21)	3.05 (3.69)	1.95 (3.96)	0.53
Emotional well-being	5.56 (4.91)	3.56 (3.98)	2.00 (4.67)	0.50
Social well-being	6.63 (6.58)	3.78 (4.99)	2.85 (5.84)	0.57
Total CPQ8-10 scores	23.07 (15.98)	15.41 (14.59)	7.66 (15.30)	0.53
*Hall Technique (n* = *64)*				
Oral symptoms	6.47 (3.99)	4.81 (3.46)	1.66 (4.86)	0.48
Function limitations	4.55 (4.35)	2.28 (2.94)	2.27 (3.71)	0.77
Emotional well-being	5.27 (5.17)	3.50 (4.73)	1.77 (4.95)	0.37
Social well-being	6.08 (6.55)	3.38 (4.62)	2.70 (5.67)	0.59
Total CPQ8-10 scores	22.36 (17.06)	13.97 (13.15)	8.39 (15.23)	0.64

*SD* standard deviation

^▲^Indicates no difference statistically

#### Teeth exfoliation

**Fig. 9:**
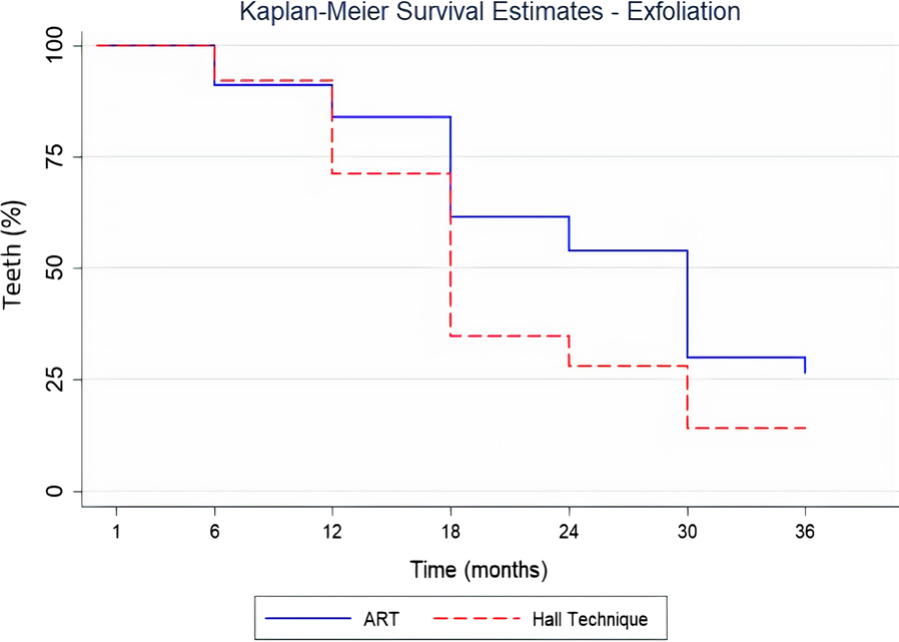
Kaplan–Meier survival curves related to tooth exfoliation for both groups (n = 125, as six teeth (4.6%) with a Major failure were not included in the analysis)

A post-hoc analysis of tooth exfoliation for both groups using Cox regression found that HT teeth exfoliated earlier than ART treated teeth (HR 1.60; *p* = 0.030; CI 1.05–2.45) (Table 6). Kaplan–Meier survival curves are shown in Fig. 9. The median time that tooth exfoliated in the ART group was 24 months (IQR = 15–30) and 18 months for the HT (IQR = 12–24).

**Table 6:** Univariate and adjusted Cox regression analysis for teeth exfoliation (n = 125)

Variable	Total n (%)	HR Univariate^†^ 95% CI^‡^	*p* value	HR Adjusted^†^ 95% CI^‡^	*p* value
*Group*					
ART (ref)	60 (92.3)				
Hall Technique	65 (98.5)	1.60 1.05–2.45	0.030*	1.84 1.19–2.87	0.007*
*Age (years)*					
5–6.9 (ref)	21 (87.5)				
7–8.9	73 (93.3)	7.75 2.79–21.5	< 0.001*	8.89 3.17–24.88	< 0.001*
≥ 9	31 (96.9)	12.62 4.36–36.51	< 0.001*	17.08 5.76–50.62	< 0.001*
*Sex*					
Male (ref)	75 (93.8)				
Female	50 (98)	0.83 0.54–1.29	0.409		
*Jaw*					
Upper (ref)	76 (96.2)				
Lower	49 (94.2)	1.00 0.65–1.53	0.994		
*Side*					
Right (ref)	66 (94.3)				
Left	59 (96.7)	0.83 0.55–1.27	0.404		
*Tooth*					
1st primary molar (ref)	76 (92.7)				
2nd primary molar	49 (100)	0.75 0.48–1.15	0.188	0.64 0.41–0.99	0.047*

^†^*HR* Hazard ratio, ^‡^*CI* confidence Interval

^*^ Indicates statistically significance differences (*p* < 0.05)

## Discussion

This randomised controlled trial seems to be novel in two respects. Firstly, ART was compared to the HT and secondly, the HT was carried out in a community setting with no access to dental facilities. The HT achieved similarly high survival rates to trials set in dental clinics (between 95 and 98% after 5 years) [29–31, 37] and equivalent to conventional crowns [38]. At 3 years, the HT had a statistically, and clinically, higher survival rate in dentinal occluso-proximal, cavitated carious lesion dentinal carious lesions in primary molars than ART (HT = 93.4%; ART = 32.7%). In other words: the HT had almost 1/10 unsuccessful restorations compared to approximately 7/10 in the ART group over 3 years.

ART was developed to be carried out without a dental chair, rotary instruments, aspiration, air-drying or radiography to observe the lesion’s depth (with cavities’ size limitations). Although studies support ART for primary teeth occlusal lesions, showing high survival rates with around 90% after 3 years [19] and an annual failure rate of approximately 5% [12, 21], the survival rates in occluso-proximal lesions are lower varying across studies; from 93 to 12% after 3 years follow-up with a mean annual failure rate from 17% [12] to 25% [21] presented by three different systematic reviews [18, 19, 21], which included results of eight different clinical trials. Investigations into the association between ART restoration failure and operators’ level of experience have shown contradictory findings [33, 39–42]. In this trial, the operators were one Specialist in Paediatric Dentistry and two Undergraduate Students, all trained in both techniques. Independent of the operators’ level of experience, restoration survival rates for the ART group were still low (32.7%) and within the wide range presented by these systematic reviews (from 93 to 12%) for restoration survival when using the ART. This also shows that ART survival rates are not as consistent as those observed with the HT.

The known side effect of a temporary OVD increase with the HT has been shown to resolve within 2–4 weeks [27, 34, 43]. Our results agree with this, showing a return to pre-treatment OVD within four weeks.

Until a few decades ago, conventional PMCs were available to paediatric dentists in Brazil. However, the conventional technique is complex, requiring local anaesthetic and tooth preparation. At the same time, less sensitive techniques and materials, especially tooth coloured materials such as GIC and resin composites, were developed. This led to a reduction in the market for dental companies selling PMCs causing discontinuation of crown availability. The higher clinical success (survival rate) of the HT than ART, means that if preformed metal crowns were available in Brazil, the HT could be a more feasible treatment option for multi-surface carious lesions in primary molars. However, aesthetics may be a concern for parents.

Patient-centred outcomes have been of growing interest, especially in paediatric dentistry [5, 14–17, 14, 15]. The largest subset of them, patient-reported outcome measures (PROMs), allows patients to give their own perceptions rather than them being gauged, and reported, by the person providing the treatment who will bring their own cognitive biases. Even an independent assessor (who is not the care provider) can be inaccurate in reporting a child’s level of discomfort. Using a child appropriate measure allowing the child to rate their experience in a ‘safe’ setting away from the care provider, is likely to give them the best opportunity to represent their feelings and thoughts most accurately.

To increase the possibility that children felt able to report their experience (positive or negative), without feeling pressured to please their dentist or being embarrassed in front of other children, they were assessed using the WBFPS immediately after treatment but out of the presence of the dentist providing the treatment and other children. Although overall discomfort scores indicated that both treatments gave low levels of discomfort, children who had a separator and a HT crown placed rated a higher discomfort level compared to those in the ART group. Both the separator and crown have to be pushed over the tooth or (for the crown) the patient can bite onto a cotton roll to push it over the tooth. Both options require a degree of pressure. Although there was a statistically significant difference between the HT and ART scores for discomfort levels reported by children, in relative terms the scores were low with over 70% of the children reporting “no” or “very low” discomfort for both timepoints for the HT group and over 80% for ART. This is similar to a trial comparing children’s discomfort between three caries management strategies where over 80% of children reported “very low” or “low” discomfort for the HT and no statistically significant differences compared to the other treatments (non-restorative treatment = 88%; conventional treatment = 72%) [27].

Children and parents’ treatment acceptability high levels were high for children, with the majority (ART = 73.9% and HT = 81.8%) answering “strongly agree” and “agree” to all questions. However, parental acceptability for appearance differed, with around a quarter (24%) of parents in the HT group but only 1 in 20 (5%) in the ART group disagreeing with the statement “the appearance of my child’s new restoration does not bother me”. ART uses glass ionomer cement, similar in colour to teeth and might not be noticed when looking in the children’s mouth whereas the metal, silver, shiny HT crown is easily noticed and especially visible if placed on a first primary molar when the child smiles or opens the mouth wide. In a study where children and their parents’ opinions on dental restorations were sought [44], 10 out of 11 parents (91%) preferred an aesthetic material (composite resin or GIC). The authors speculate that this might be related to a concern of the parents regarding a visible sign of perceived “lack of care”. The obvious appearance of the silver crown did not seem to concern children. Only 5 (8%) (ART = 2; HT = 3) said they would not show their treated tooth to their friends. Although young children may be less aware of, or bothered by, the aesthetics of their teeth than older children or adults, they may also have different aesthetic ‘norms’ compared to their parents. There were no other differences in parent’s/carers’ opinions of the treatments, in common with another study [27].

Children had only one tooth treated and included in this trial and if they needed further dental treatments, they were referred to the public dental service. This is a potential limitation of the trial as treatment may have influenced children’s OHRLQoL and may explain the improvement in both groups’ OHRQoL at 6-month follow-up despite only a single tooth being treated. In a similar setting, ART treatment of carious lesions was found to lead to a greater improvement in children’s OHRQoL when compared to caries-free children [45]. The authors suggest that the children’s positive perception of their dental care might have influenced their OHRQoL.

The study was carried out in Brazilian children and this might also pose a limitation for generalising this study results for the secondary outcomes. As cultural differences are often present in different populations, the self-reported discomfort and treatment acceptance by children and their parents may differ if treatments are applied in a different setting, communities, population or culture.

Since this trial was conducted in the schools with no access to dental facilities, radiographs could not be performed and the real extension of the lesions could not be assessed. When considering a dental setting, it is very likely that a more accurate diagnosis related to cavities depth would be performed. Likewise, the dental setting would provide better resources as lighting and equipment for performing the restorations and it is unlikely the outcomes would be worse than the presented in this manuscript.

The protocol for this trial had not previously set out to investigate tooth exfoliation. However, early exfoliation of teeth treated with the HT was observed. This differed from a retrospective study evaluating the same outcome where no difference was observed within the same child [46]. Further information related to the contralateral teeth (if present/absent) was not collected when the study tooth was evaluated by the outcome assessor.

The clinical success and low re-treatment rate of the HT might influence parental acceptability and outweighed concerns over appearance. This change in acceptability has been found in a study weighing up the disadvantages of discolouration of teeth using silver diamine fluoride with other treatments [47]. Parents’ “tipping point” for accepting discolouration changed when faced with other options they considered less favourable (e.g. sedation or general anaesthesia).

Shared clinical decision-making allows the clinician and patient (or parents/carers) to reach an informed decision. Treatment choices to manage carious lesions for children are not only based on which is the most comfortable, but also consider effectiveness and acceptability, for both children and their parents.

In Brazil, ART is the treatment of choice for children outside the clinical setting as no clinical facilities or complex devices are required. It is also commonly used in the public health service, because of its low resource costs, both for material and clinician time. Although this trial was conducted in Brazil, its results can be extrapolated worldwide given that the HT has shown similarly high success rates in clinical and non-clinical settings, where the conventional clinical facilities are not available.

Similar to ART, the HT is a non-aerosol generating procedure (non-AGP), so is particularly suitable as a treatment option during outbreaks of highly infectious diseases, such as COVID-19, when dental health care workers are potentially at high risk of infection from infected aerosolisation and droplet spread of contaminated body fluids.

## Conclusion

Discomfort scores were lower for ART treated teeth, although were within acceptable clinical limits for both groups. There was high acceptability from parents and children for both treatments. However, a higher proportion of parents were bothered by the appearance of the HT crowns compared to the ART fillings. Both treatments are applicable where dental facilities are not available, being minimally invasive approaches, and non-AGPs, reducing cross-infection risk of dental treatment from aerosols and droplets. However, the HT restoration survival rate was almost three times higher than ART (93.4% compared to 32.7%) for restoring occluso-proximal dentine lesions in primary molars after 3 years.

## Supplementary information

**Additional file 1**. Protocol for restoring occluso-proximal lesions using the Atraumatic Restorative Treatment (ART) [32].

**Additional file 2**. Protocol for restoring occluso-proximal lesions using the Hall Technique (HT) [32].

**Additional file 3**. Treatment acceptability questionnaire (children).

**Additional file 4**. Treatment acceptability questionnaire (parents/carers).

**Additional file 5**. Participants’ baseline characteristics.

**Additional file 6**. Schedule of outcome assessments.

**Additional file 7**. Survival rate for both arms at each timepoint for ART and HT groups (n = 131).

**Additional file 8**. Ordered Logistic Regression analysis of the discomfort scores after treatment between the groups and the independent variables (considering only the discomfort scores after crown placement).

## Data Availability

The datasets used and/or analysed during the current study are available from the corresponding author on reasonable request.

## Abbreviations

ART: Atraumatic Restorative Treatment
CI: Confidence interval
COVID-19: Coronavirus Disease 2019
CPQ8-10: Child Perception Questionnaire (8–10 years)
DMFT: Decayed missing filled teeth (permanent dentition)
dmft: Decayed missing filled teeth (primary dentition)
GIC: Glass ionomer cement
HR: Hazard ratio
HT: Hall Technique
IQR: Interquartile range
IRR: Incidence rate ratio
MID: Minimally invasive dentistry
Non-AGPs: Non-aerosol generating procedures
OHRQoL: Oral Health Related Quality of Life
OR: Odds ratio
OVD: Occlusal vertical dimension
PMC: Preformed metal crown
PROMs: Patient-Reported Outcome Measures
SD: Standard deviation
SE: Standard error
SDCEP: Scottish Dental Clinical Effectiveness Programme
WBFPS: Wong-Baker Faces Pain Scale

## References

[1] Brasil. Ministério da Saúde. SB Brasil 2010: Pesquisa nacional de saúde bucal—resultados principais. Brasília, DF: Ministério da Saúde; 2012. https://bvsms.saude.gov.br/bvs/publicacoes/pesquisa_nacional_saude_bucal.pdf. Accessed 15th Nov 2019.

[2] Ricketts DNJ, Pitts NB (2009). Traditional operative treatment options. Monogr Oral Sci.

[3] BaniHani A, Deery C, Toumba J, Munyombwe T, Duggal M (2018). The impact of dental caries and its treatment by conventional or biological approaches on the oral health-related quality of life of children and carers. Int J Paediatr Dent.

[4] Cunnion DT, Spiro A, Jones JA, Rich SE, Papageorgiou CP, Tate A, Casamassimo P, Hayes C, Garcia RI (2010). Pediatric oral health-related quality of life improvement after treatment of early childhood caries: a prospective multisite study. J Dent Child (Chic).

[5] Klingberg G, Broberg AG (2007). Dental fear/anxiety and dental behaviour management problems in children and adolescents: a review of prevalence and concomitant psychological factors. Int J Paediatr Dent.

[6] Gao X, Hamzah SH, Yiu CK, McGrath C, King NM (2013). Dental fear and anxiety in children and adolescents: qualitative study using YouTube. J Med Internet Res.

[7] Ladewig NM, Tedesco TK, Gimenez T, Braga MM, Raggio DP (2018). Patient-reported outcomes associated with different restorative techniques in pediatric dentistry: a systematic review and MTC meta-analysis. PLoS ONE.

[8] Alshoraim MA, El-Housseiny AA, Farsi NM, Felemban OM, Alamoudi NM, Alandejani AA (2018). Effects of child characteristics and dental history on dental fear: cross-sectional study. BMC Oral Health.

[9] van Bochove JA, van Amerongen WE (2006). The influence of restorative treatment approaches and the use of local analgesia, on the children's discomfort. Eur Arch Paediatr Dent.

[10] Schwendicke F, Frencken JE, Bjørndal L, Maltz M, Manton DJ, Ricketts D, Van Landuyt K, Banerjee A, Campus G, Doméjean S, Fontana M, Leal S, Lo E, Machiulskiene V, Schulte A, Splieth C, Zandona AF, Innes NP (2016). Managing carious lesions: consensus recommendations on carious tissue removal. Adv Dent Res.

[11] Frencken JE, Peters MC, Manton DJ, Leal SC, Gordan VV, Eden E (2012). Minimal intervention dentistry for managing dental caries—a review: report of a FDI task group. Int Dent J.

[12] Frencken JE (2017). Atraumatic restorative treatment and minimal intervention dentistry. Br Dent J.

[13] Frencken JE, Pilot T, Songpaisan Y, Phantumvanit P (1996). Atraumatic restorative treatment (ART): rationale, technique, and development. J Public Health Dent..

[14] Farag A, Frencken JE (2009). Acceptance and discomfort from atraumatic restorative treatment in secondary school students in Egypt. Med Princ Pract.

[15] de Menezes Abreu DM, Leal SC, Frencken JE (2009). Self-report of pain in children treated according to the atraumatic restorative treatment and the conventional restorative treatment—a pilot study. J Clin Pediatr Dent..

[16] Rahimtoola S, van Amerongen E, Maher R, Groen H (2000). Pain related to different ways of minimal intervention in the treatment of small caries lesions. ASDC J Dent Child..

[17] Schriks MC, van Amerongen WE (2003). Atraumatic perspectives of ART: psychological and physiological aspects of treatment with and without rotary instruments. Community Dent Oral Epidemiol.

[18] Tedesco TK, Gimenez T, Floriano I, Montagner AF, Camargo LB, Calvo AFB, Morimoto S, Raggio DP (2018). Scientific evidence for the management of dentin caries lesions in pediatric dentistry: a systematic review and network meta-analysis. PLoS ONE.

[19] de Amorim RG, Frencken JE, Raggio DP, Chen X, Hu X, Leal SC (2018). Survival percentages of atraumatic restorative treatment (ART) restorations and sealants in posterior teeth: an updated systematic review and meta-analysis. Clin Oral Investig.

[20] Frencken JE, Van't Hof MA, Van Amerongen WE, Holmgren CJ (2004). Effectiveness of single-surface ART restorations in the permanent dentition: a meta-analysis. J Dent Res..

[21] Ruengrungsom C, Palamara JEA, Burrow MF (2018). Comparison of ART and conventional techniques on clinical performance of glass-ionomer cement restorations in load bearing areas of permanent and primary dentitions: a systematic review. J Dent.

[22] Tedesco TK, Calvo AF, Lenzi TL, Hesse D, Guglielmi CA, Camargo LB, Gimenez T, Braga MM, Raggio DP (2017). ART is an alternative for restoring occlusoproximal cavities in primary teeth—evidence from an updated systematic review and meta-analysis. Int J Paediatr Dent.

[23] Innes NP, Stirrups DR, Evans DJ, Hall N, Leggate M (2006). A novel technique using preformed metal crowns for managing carious primary molars in general practice—a retrospective analysis. Br Dent J..

[24] Innes N, Evans D, Stewart M, Keightley A. The Hall Technique: A minimal intervention, child centred approach to managing the carious primary molar. A Users Manual, Version 4. https://upload.wikimedia.org/wikipedia/commons/9/91/HallTechGuide_V4.pdf. Dundee: University of Dundee; 2015. Accessed 15th Nov 2019.

[25] American Association of Pediatric Dentistry. Pediatric Restorative Dentistry: reference manual. Pediatr Dent 2018–2019; 40(6):330–42. https://www.aapd.org/globalassets/media/policies_guidelines/bp_restorativedent.pdf?v=new. Accessed 15th Nov 2019.

[26] Scottish Dental Effectiveness Programme (SDCEP). Prevention and management of dental caries in children: dental clinical guidance 2nd edition. 2018; https://www.sdcep.org.uk/wp-content/uploads/2018/05/SDCEP-Prevention-and- Management-of-Dental-Caries-in-Children-2nd-Edition.pdf. Accessed 15th Nov 2019.

[27] Santamaria RM, Innes NP, Machiulskiene V, Evans DJ, Alkilzy M, Splieth CH (2015). Acceptability of different caries management methods for primary molars in a RCT. Int J Paediatr Dent.

[28] Bell SJ, Morgan AG, Marshman Z, Rodd HD (2010). Child and parental acceptance of preformed metal crowns. Eur Arch Paediatr Dent.

[29] Innes NP, Evans DJ, Stirrups DR (2007). The Hall Technique; a randomized controlled clinical trial of a novel method of managing carious primary molars in general dental practice: acceptability of the technique and outcomes at 23 months. BMC Oral Health.

[30] Santamaria RM, Innes NP, Machiulskiene V, Evans DJ, Splieth CH (2014). Caries management strategies for primary molars: 1-yr randomized control trial results. J Dent Res.

[31] Innes NP, Evans DJ, Stirrups DR (2011). Sealing caries in primary molars: randomized control trial, 5-year results. J Dent Res.

[32] Hesse D, de Araujo MP, Olegário IC, Innes N, Raggio DP, Bonifácio CC (2016). Atraumatic Restorative Treatment compared to the Hall Technique for occluso-proximal cavities in primary molars: study protocol for a randomized controlled trial. Trials.

[33] de Amorim RG, Leal SC, Frencken JE (2012). Survival of atraumatic restorative treatment (ART) sealants and restorations: a meta-analysis. Clin Oral Investig.

[34] van der Zee V, van Amerongen WE (2010). Short communication: Influence of preformed metal crowns (Hall Technique) on the occlusal vertical dimension in the primary dentition. Eur Arch Paediatr Dent.

[35] Wong DL, Baker CM (1988). Pain in children: comparison of assessment scales. Pediatr Nurs.

[36] Barbosa TS, Tureli MC, Gavião MB (2009). Validity and reliability of the Child Perceptions Questionnaires applied in Brazilian children. BMC Oral Health.

[37] Santamaria RM, Innes NPT, Machiulskiene V, Schmoeckel J, Alkilzy M, Splieth CH (2018). Alternative caries management options for primary molars: 2.5-year outcomes of a randomised clinical trial. Caries Res.

[38] Elamin F, Abdelazeem N, Salah I, Mirghani Y, Wong F (2019). A randomized clinical trial comparing Hall vs conventional technique in placing preformed metal crowns from Sudan. PLoS ONE.

[39] Kemoli AM, van Amerongen WE, Opinya G (2009). Influence of the experience of operator and assistant on the survival rate of proximal ART restorations: two-year results. Eur Arch Paediatr Dent.

[40] Frencken JE, Makoni F, Sithole WD, Hackenitz E (1998). Three-year survival of one-surface ART restorations and glass-ionomer sealants in a school oral health programme in Zimbabwe. Caries Res.

[41] Olegário IC, Pacheco AL, de Araújo MP, Ladewig NM, Bonifácio CC, Imparato JC, Raggio DP (2017). Low-cost GICs reduce survival rate in occlusal ART restorations in primary molars after one year: a RCT. J Dent.

[42] Bonifácio CC, Hesse D, Bönecker M, Van Loveren C, Van Amerongen WE, Raggio DP (2013). A preliminary clinical trial using flowable glass-ionomer cement as a liner in proximal-ART restorations: the operator effect. Med Oral Patol Oral Cir Bucal.

[43] So D, Blain K, Innes N, Evans D, Borrie F, Roughley M, Lamont T, Keightley A, Gardner A, Hussein I, De Souza N. Measurement of occlusal equilibration following Hall crown Placement; pilot study. J Dent Res. 2015; 94 (Spec Issue A) Abstract 0080, (www.iadr.org).

[44] Maciel R, Salvador D, Azoubel K, Redivivo R, Maciel C, da Franca C, Amerongen E, Colares V (2017). The opinion of children and their parents about four different types of dental restorations in a public health service in Brazil. Eur Arch Paediatr Dent.

[45] Paula JS, Tôrres LH, Ambrosano GM, Mialhe FL (2012). Association between oral health-related quality of life and atraumatic restorative treatment in school children: an exploratory study. Indian J Dent Res.

[46] Araujo MP, Uribe S, Robertson MD, Mendes FM, Raggio DP, Innes NPT (2020). The Hall Technique and exfoliation of primary teeth: a retrospective cohort study. Br Dent J.

[47] Crystal YO, Janal MN, Hamilton DS, Niederman R (2017). Parental perceptions and acceptance of silver diamine fluoride staining. J Am Dent Assoc.

