# Supplementary material for: Atraumatic restorative treatment compared to the Hall Technique for occluso-proximal carious lesions in primary molars; 36-month follow-up of a randomised control trial in a school setting
Source: BMC Oral Health. 2020 Nov 11;20:318. doi: 10.1186/s12903-020-01298-x (PMC7656501; doi:10.1186/s12903-020-01298-x)
Supplement: Supplementary file 1 — Additional file 1. Protocol for restoring occluso-proximal lesions using the Atraumatic Restorative Treatment (ART) [32]. [file 12903_2020_1298_MOESM1_ESM.docx]

**Additional file 1 –** Protocol for restoring occluso-proximal lesions using the Atraumatic Restorative Treatment (ART) [32].

| 1. Preparing the cavity | • Place cotton wool rolls alongside the tooth to be treated.  • Remove plaque from tooth surface with wet cotton wool pellets.  • Dry the tooth surface with dry cotton wool pellets.  • If necessary, make the entrance of the cavity wider with a dental hatchet.  • Remove the carious dentine with excavators, until the enamel-dentine junction is caries free.  • Fracture off unsupported thin enamel with the hatchet.  • Clean the cavity with wet and then dry with cotton wool pellets.  • Remove the caries near the pulp carefully. In this area only the completely soft and demineralized tissue must be removed.  • Clean the cavity again with wet cotton wool pellets.  • Complete the procedure by drying the cavity with dry cotton wool pellets.  • Place a matrix strip between the teeth. This matrix must be pre-curved.  • Insert a wedge to support the strip under the contact point at the gum margin. Advise the child that he/she might feel a little uncomfortable during this procedure. |
| --- | --- |
| 2. Conditioning the cavity | • Apply one drop of GC Cavity Conditioner liquid (GC Europe, Leuven, Belgium) on paper pad.  • Dip a cotton wool pellet in clean water.  • Remove excess of water from the cotton wool pellet by lightly touching  against a dry cotton wool roll, tissue or gauze.  • Dip the moist cotton wool pellet in the GC Cavity Conditioner liquid.  • Condition the cavity and adjacent fissures with the liquid for 10–15 seconds.  • Wash the cavity and fissures immediately with three sequences of cotton wool pellets, dipped in clean water.  • Dry the cavity with three sequences of dry cotton wool pellets. |
| 3. Restoring the cavity | • Ensure that the tooth is kept dry during the restoration phase – if moisture could not be maintained, repeat Step 2.  • The capsule of EQUIA Forte, capsules (GC Europe, Leuven, Belgium) should be activated and mixed. Before activation, shake the capsule or tap its side on a hard surface to loosen the powder. To activate the capsule, push the plunger until it is flush with the main body. Immediately place the capsule into a metal capsule applier and click the lever once and it will be activated. Immediately remove the capsule and set it into a mixer (or an amalgamator) and mix for 10 seconds  • Remove the capsule from mixer and place into a metal capsule applier again. Make two clicks to prime the capsule. Within 10 seconds maximum after mixing, start to insert the mixture directly into the prepared cavity.  • Overfill the cavity extending to pits and fissures. Extra care should be taken to avoid moisture contamination or drying out.  • Place the index finger over the restorative material and press the material to accommodate to the cavity  • Remove finger sideways after a few seconds  • Remove visible excesses of glass ionomer cement (GIC) with a medium or large excavator  • Wait 1–2 minutes till the material feels hard, whilst keeping the tooth dry  • Remove matrix and wedge carefully turning the matrix the other side (adjacent tooth) and check the bite using articulation paper  • Adjust the height of the restoration with the carver if necessary  • Using a micro-tip applicator, apply G-Coat Plus (GC Europe, Leuven, Belgium) to the occlusal and proximal surface (using a new metallic matrix band embedded with the coating and a new wedge) of the restoration.  • Light cure for 20 seconds at occlusal, 20 seconds at buccal and 20 seconds at the lingual surface. G-Coat Plus is indicated to seal and protect the surface of glass ionomer  • Remove the matrix, wedge and cotton wool rolls  • Child will be told not to eat for at least 1 hour after the restoration was performed. A sticker with the time at which they are allowed to eat again was placed on their t-shirt |
