# Supplementary material for: Atraumatic restorative treatment compared to the Hall Technique for occluso-proximal carious lesions in primary molars; 36-month follow-up of a randomised control trial in a school setting
Source: BMC Oral Health. 2020 Nov 11;20:318. doi: 10.1186/s12903-020-01298-x (PMC7656501; doi:10.1186/s12903-020-01298-x)
Supplement: Supplementary file 2 — Additional file 2. Protocol for restoring occluso-proximal lesions using the Hall Technique (HT) [32]. [file 12903_2020_1298_MOESM2_ESM.docx]

**Additional file 2 –** Protocol for restoring occluso-proximal lesions using the Hall Technique (HT) [32].

| First visit | • Assess the tooth shape, contact points/areas and child’s occlusion.  • Use orthodontic separators to create space for fitting the Hall crown, unless there are no contact points. In order to protect the airways, the child should be sat upright  • Thread two lengths of dental floss through the separator. Stretch the separator and floss taught and floss through the contact point briskly and firmly until the leading edge only is felt “popping through” the contact point. Remove the floss and make a second appointment with the patient 3 to 5 days later. |
| --- | --- |
| Second visit  (3 to 5 days after the first visit) | • Remove the separator with an excavator.  • Gently remove loose plaque and food debris only from the cavity.  • Assess the occlusion: measure the patient’s occlusal-vertical dimension (OVD) with a millimetre probe using the distance between the most coronal points of the primary canines in order to assess the degree of overbite after crown’s placement.  • Protect child’s airways by placing a gauze swab square between the tongue and the tooth to be crowned.  • Select the correct crown size (Stainless Steel Crowns, 3M™ ESPE™, St. Paul, MN, USA). The crown should cover all the cusps and reach the contact points, with a slight feeling of “spring back.” You should aim to fit the smallest size of crown which will seat.  • Keep the treatment area free from saliva by isolating the tooth with cotton wool rolls.  • Dry the inside of the crown with dry cotton pellets.  • Mix the encapsulated glass ionomer cement (GIC) (Fuji I, GC Europe, Leuven, Belgium) for 10 seconds, according to the manufacturer’s instructions.  • Load the crown generously with GIC (at least two thirds full). Start from the bottom to the top of the crown to avoid air blows and voids.  • Place the crown over the tooth and seat the crown into place by finger pressure or ask the child to bite it into place.  • Check the crown position as soon the crown is fitted.  • Wipe away the excess GIC with a cotton wool roll or the gauze swab used to protect the airway.  • Place a cotton wool roll between the crown and the opposing tooth and ask the child to bite firmly on the crown for another 2–3 minutes.  • Remove excess cement, flossing between the contacts.  • Measure child’s OVD and record in the notes. If excessive, then consider removing the entire crown (mean OVD difference 1.5mm)  • Gingival blanching usually disappears within minutes. The occlusal discrepancy should resolve in a few weeks.  • Check the buccal relationship of the crowned tooth with its opposing number. If there is a displacing contact, resulting in a cross bite, then manage as for excessive bite propping. |
