# Supplementary material for: Atraumatic restorative treatment compared to the Hall Technique for occluso-proximal carious lesions in primary molars; 36-month follow-up of a randomised control trial in a school setting
Source: BMC Oral Health. 2020 Nov 11;20:318. doi: 10.1186/s12903-020-01298-x (PMC7656501; doi:10.1186/s12903-020-01298-x)
Supplement: Supplementary file 3 — Additional file 3. Treatment acceptability questionnaire (children). [file 12903_2020_1298_MOESM3_ESM.docx]

**Additional file 3** – Treatment acceptability questionnaire (children)

|  | 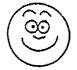  Strongly agree | 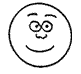  Agree | 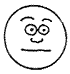  No opinion | 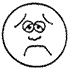  Disagree | 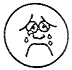  Strongly disagree |
| --- | --- | --- | --- | --- | --- |
| 1. Are you happy with the tooth you have had fixed? |  |  |  |  |  |
| 2. Are you going to show your fixed tooth to your friends? |  |  |  |  |  |
| 3. Did you think the dentist treated you well? |  |  |  |  |  |
| 4. Did you understand everything the dentist was going to do to your tooth? |  |  |  |  |  |
| 5. How happy would you be if people asked to see the tooth you have had fixed? |  |  |  |  |  |

.
