# Supplementary material for: Atraumatic restorative treatment compared to the Hall Technique for occluso-proximal carious lesions in primary molars; 36-month follow-up of a randomised control trial in a school setting
Source: BMC Oral Health. 2020 Nov 11;20:318. doi: 10.1186/s12903-020-01298-x (PMC7656501; doi:10.1186/s12903-020-01298-x)
Supplement: Supplementary file 4 — Additional file 4. Treatment acceptability questionnaire (parents/carers). [file 12903_2020_1298_MOESM4_ESM.docx]

**Additional file 4** – Treatment acceptability questionnaire (parents/carers)

|  | Strongly Agree | Agree | No Opinion | Disagree | Strongly Disagree |
| --- | --- | --- | --- | --- | --- |
| **1. I understood the reason why my child needed a restoration** |  |  |  |  |  |
| **2. The appearance of my child’s new restoration does not bother me.** |  |  |  |  |  |
| **3. I think my child’s new restoration is really protecting his/her tooth.** |  |  |  |  |  |
| **4. I believe that my child felt good during the treatment carried out.** |  |  |  |  |  |
| **5. I believe that the dental team was nice and helpful during my child’s treatment.** |  |  |  |  |  |
