# Supplementary material for: Atraumatic restorative treatment compared to the Hall Technique for occluso-proximal carious lesions in primary molars; 36-month follow-up of a randomised control trial in a school setting
Source: BMC Oral Health. 2020 Nov 11;20:318. doi: 10.1186/s12903-020-01298-x (PMC7656501; doi:10.1186/s12903-020-01298-x)
Supplement: Supplementary file 5 — Additional file 5. Participants’ baseline characteristics. [file 12903_2020_1298_MOESM5_ESM.docx]

**Additional file 5 –** Participants’ baseline characteristics.

|  | | **ART** | **Hall Technique** | ***p*-value** | **Total** |
| --- | --- | --- | --- | --- | --- |
| **Age  (years)** | Mean (SD) | 7.98 (±1.07) | 8.21 (±1.22) | 0.254 ▲ | 8.1 (±1.15) |
|  | Minimum | 5 | 6 |  | 5 |
|  | Maximum | 10 | 10 |  | 10 |
| **Sex**  **n (%)** | Male | 39 (60) | 41 (62) | 0.804 ‡ | 80 (61) |
|  | Female | 26 (40) | 25 (38) |  | 51 (39) |
| **dmft/DMFT n (%)** | 1-2 | 20 (31) | 27 (41)* | 0.253 ‡ | 47 (36) |
|  | 3-4 | 22 (34) | 23 (35)* |  | 45 (34) |
|  | ≥5 | 23 (35) | 15 (23)* |  | 38 (29) |
| **Tooth n (%)** | Upper first primary molar (54/64) | 19 (29) | 17 (26) | 0.557 ‡ | 36 (27) |
|  | Upper second primary molar (55/65) | 24 (37) | 19 (29) |  | 43 (33) |
|  | Lower first primary molar (74/84) | 19 (29) | 27 (41) |  | 46 (35) |
|  | Lower second primary molar (75/85) | 3 (5) | 3 (4) |  | 6 (5) |
| ▲= t-test  ‡ = chi-square test  *One child in the HT group did not have dmft/DMFT collected by the operators at the baseline  **One child in the ART group did not have Plaque and Gingival index collected at the baseline | | | | | |
