# Supplementary material for: Atraumatic restorative treatment compared to the Hall Technique for occluso-proximal carious lesions in primary molars; 36-month follow-up of a randomised control trial in a school setting
Source: BMC Oral Health. 2020 Nov 11;20:318. doi: 10.1186/s12903-020-01298-x (PMC7656501; doi:10.1186/s12903-020-01298-x)
Supplement: Supplementary file 6 — Additional file 6. Schedule of outcome assessments. [file 12903_2020_1298_MOESM6_ESM.docx]

**Additional file 6 –** Schedule of outcome assessments.

| **Outcomes** |  | **Timepoints** | | | | | | | | | | | |
| --- | --- | --- | --- | --- | --- | --- | --- | --- | --- | --- | --- | --- | --- |
|  |  | **Baseline** | **Immediately after the treatment** | **1 week** | **2 weeks** | **3 weeks** | **1 month** | **6 months** | **12 months** | **18 months** | **24 months** | **30 months** | **36 months** |
| **Clinical Outcomes** |  |  |  |  |  |  |  |  |  |  |  |  |  |
| **Restoration survival** |  |  |  |  |  |  | X | X | X | X | X | X | X |
| **Occlusal Vertical Dimension (OVD)** |  | X | X | X | X | X | X |  |  |  |  |  |  |
| **Tooth exfoliation** |  |  |  |  |  |  | X | X | X | X | X | X | X |
| **Patient Reported Outcomes Measurements (PROMs)** | | | |  |  |  |  |  |  |  |  |  |  |
| **Child’s OHRQoL** |  | X |  |  |  |  |  | X |  |  |  |  |  |
| **Child self-reported discomfort** |  | X | X |  |  |  |  |  |  |  |  |  |  |
| **Acceptability of the treatment (children)** |  |  | X |  |  |  |  |  |  |  |  |  |  |
| **Acceptability of the treatment (parents)** |  |  | X |  |  |  |  |  |  |  |  |  |  |
