# Supplementary material for: Atraumatic restorative treatment compared to the Hall Technique for occluso-proximal carious lesions in primary molars; 36-month follow-up of a randomised control trial in a school setting
Source: BMC Oral Health. 2020 Nov 11;20:318. doi: 10.1186/s12903-020-01298-x (PMC7656501; doi:10.1186/s12903-020-01298-x)
Supplement: Supplementary file 7 — Additional file 7. Survival rate for both arms at each timepoint for ART and HT groups (n = 131). [file 12903_2020_1298_MOESM7_ESM.docx]

**Additional file 7 –** Survival rate for both arms at each timepoint for ART and HT groups (n=131).

| **Restoration survival rate* (SE)**  **95%CI** | | | | | | | |
| --- | --- | --- | --- | --- | --- | --- | --- |
|  | **Timepoint (months)** | | | | | | |
|  | **1** | **6** | **12** | **18** | **24** | **30** | **36** |
| **ART** | 91% (±0.04)  0.81-0.96 | 63% (±0.06);  0.49-0.74 | 52% (±0.07)  0.38-0.64 | 40% (±0.07)  0.26-0.54 | 37% (±0.07)  0.23-0.51 | 32% (±0.08)  0.17-0.47 | 32% (±0.08)  0.17-0.47 |
| **HT** | 100% | 98% (±0.02)  0.88-0.99 | 98% (±0.02)  0.88-0.99 | 93% (±0.05)  0.72-0.98 | 93% (±0.05)  0.72-0.98 | 93% (±0.05)  0.72-0.98 | 93% (±0.05)  0.72-0.98 |
| * Calculated using logrank test | | | | | | | |
