# Supplementary material for: Atraumatic restorative treatment compared to the Hall Technique for occluso-proximal carious lesions in primary molars; 36-month follow-up of a randomised control trial in a school setting
Source: BMC Oral Health. 2020 Nov 11;20:318. doi: 10.1186/s12903-020-01298-x (PMC7656501; doi:10.1186/s12903-020-01298-x)
Supplement: Supplementary file 8 — Additional file 8. Ordered Logistic Regression analysis of the discomfort scores after treatment between the groups and the independent variables (considering only the discomfort scores after crown placement). [file 12903_2020_1298_MOESM8_ESM.docx]

**Additional file 8 –** Ordered Logistic Regression analysis of the discomfort scores after treatment between the groups and the independent variables (considering only the discomfort scores after crown placement).

| **Variables** | **Unadjusted OR  (95% CI)** | ***p-*value** | **Adjusted OR  (95% CI)** | ***p-*value** |
| --- | --- | --- | --- | --- |
| **Treatment** |  |  |  |  |
| **ART (ref)** |  |  |  |  |
| **HT** | 1.95  (0.99 to 3.87) | 0.055 | 2.26  (1.11 to 4.62) | 0.025* |
| **Age (years)** |  |  |  |  |
| **5 to 6.9 (ref)** |  |  |  |  |
| **7 to 8.9** | 0.47  (0.19 to 1.16) | 0.102 | 0.50  (0.20 to 1.25) | 0.102 |
| **≥ 9** | 0.74  (0.27 to 2.00) | 0.550 | 0.62  (0.22 to 1.76) | 0.368 |
| **Sex** |  |  |  |  |
| **Male (ref)** |  |  |  |  |
| **Female** | 1.05  (0.53 to 2.09) | 0.891 |  |  |
| **Operator** |  |  |  |  |
| **Specialist (ref)** |  |  |  |  |
| **Student 1** | 0.83  (0.36 to 1.93) | 0.661 |  |  |
| **Student 2** | 1.61  (0.70 to 3.69) | 0.259 |  |  |
| **Jaw** |  |  |  |  |
| **Upper (ref)** |  |  |  |  |
| **Lower** | 1.74  (0.88 to 3.44) | 0.112 |  |  |
| **Primary Tooth** |  |  |  |  |
| **1^st^ Molar (ref)** |  |  |  |  |
| **2^nd^ Molar** | 0.49  (0.24 to 1.01) | 0.052 |  |  |
| **DMFT/dmft** |  |  |  |  |
| **0 and 1 (ref)** |  |  |  |  |
| **3 and 4** | 1.00  (0.46 to 2.18) | 0.997 | 1.02  (0.46 to 2.29) | 0.955 |
| **More than 4** | 0.42  (0.17 to 1.03) | 0.058 | 0.39  (0.15 to 1.01) | 0.052 |
| ART = Atraumatic Restorative Treatment; HT = Hall technique  OR = Odds Ratio; 95% CI = 95% Confidence Interval  * Statistically significant difference (p < 0.05) | | | | |
